# Fructose-2,6-Bisphosphate synthesis by 6-Phosphofructo-2-Kinase/Fructose-2,6-Bisphosphatase 4 (PFKFB4) is required for the glycolytic response to hypoxia and tumor growth

**DOI:** 10.18632/oncotarget.2213

**Published:** 2014-07-13

**Authors:** Jason Chesney, Jennifer Clark, Alden C. Klarer, Yoannis Imbert-Fernandez, Andrew N. Lane, Sucheta Telang

**Affiliations:** ^1^ James Graham Brown Cancer Center, Departments of Medicine (Hematology/Oncology), Pediatrics and Biochemistry and Molecular Biology, University of Louisville, Louisville, KY; ^2^ Current address: Markey Cancer Center, University of Kentucky, Lexington, KY

**Keywords:** Glycolysis, 6-Phosphofructo-2-Kinase, Fructose-2,6-Bisphosphatase, Prostate Cancer, Lung Cancer

## Abstract

Fructose-2,6-bisphosphate (F2,6BP) is a shunt product of glycolysis that allosterically activates 6-phosphofructo-1-kinase (PFK-1) resulting in increased glucose uptake and glycolytic flux to lactate. The F2,6BP concentration is dictated by four bifunctional 6-phosphofructo-2-kinase/fructose-2,6-bisphosphatases (PFKFB1-4) with distinct kinase:phosphatase activities. PFKFB4 is over-expressed in human cancers, induced by hypoxia and required for survival and growth of several cancer cell lines. Although PFKFB4 appears to be a rational target for anti-neoplastic drug development, it is not clear whether its kinase or phosphatase activity is required for cancer cell survival. In this study, we demonstrate that recombinant human PFKFB4 kinase activity is 4.3-fold greater than its phosphatase activity, siRNA and genomic deletion of *PFKFB4* decrease F2,6BP, PFKFB4 over-expression increases F2,6BP and selective PFKFB4 inhibition *in vivo* markedly reduces F2,6BP, glucose uptake and ATP. Last, we find that PFKFB4 is required for cancer cell survival during the metabolic response to hypoxia, presumably to enable glycolytic production of ATP when the electron transport chain is not fully operational. Taken together, our data indicate that the PFKFB4 expressed in multiple transformed cells and tumors functions to synthesize F2,6BP. We predict that pharmacological disruption of the PFKFB4 kinase domain may have clinical utility for the treatment of human cancers.

## INTRODUCTION

Glucose metabolism is regulated by a family of four bifunctional 6-phosphofructo-2-kinase/ fructose-2,6-bisphosphatases (PFKFB1-4) that determine the concentration of fructose 2,6-bisphosphate (F2,6BP), which is a potent allosteric activator of the glycolytic enzyme 6-phosphofructo-1-kinase (PFK-1) [1]. Whereas F2,6BP activates PFK-1, several metabolic products including ATP, H+ ions and citrate, allosterically inhibit PFK-1, indicating that PFK-1 is an essential metabolic sensor in the glycolytic pathway [2]. Given the central role that the PFKFB family members play in regulating glucose metabolism via PFK-1, understanding their specific functions within cells and tissues is of paramount importance to the study of a multitude of disease states caused by inflammation, ischemia and neoplastic transformation.

Within five years of the initial discovery of F2,6BP in 1980 [3, 4], multiple PFKFB enzyme activities were detected in several organs that were distinguished by having markedly reduced but still detectable fructose-2,6-bisphosphatase activities relative to the liver PFKFB1 family member which has a near 1:1 kinase:bisphosphatase ratio (1). Interestingly, a distinct PFKFB activity was detected in the testes [5] and a human testes-specific *PFKFB4* gene then was cloned in 1999 [6] suggesting that the unique metabolic requirements of sperm may necessitate a divergent PFKFB family member. Although several reports indicated that the testes PFKFB4 from various species display approximately 3-5 fold higher kinase than phosphatase activity [5, 7, 8], suggesting that its function was predominantly to increase glycolytic flux into the 3-carbon portion of the pathway, one study of purified protein reported a near 1:1 ratio similar to that of the liver PFKFB1 family member [9].

In the early 2000s, Minchenko *et al.* demonstrated that the testes PFKFB4 was induced by hypoxia in multiple cancer cell lines and over-expressed in matched human lung, breast and colon tumor tissues relative to normal tissues from the same patients [10-13]. The functional requirement of PFKFB4 for tumor growth then was published in 2010 when researchers demonstrated that selective inhibition of PFKFB4 with siRNA suppressed the growth of human lung adenocarcinoma xenografts in athymic mice (U.S. Patent #8,283,332; PFKFB4 Inhibitors and Methods of Using the Same). The importance of PFKFB4 as a potential target for the development of cancer therapeutics was then significantly expanded in 2012 by two independent groups that conducted unbiased screens for genes essential for cancer survival and found that PFKFB4 is required for both glioma stem-like cell [14] and prostate cancer cell survival [15] but not for normal cell survival. Taken together, these studies indicated that PFKFB4 may be a useful molecular target for the development of anti-neoplastic agents.

An essential first step in developing anti-cancer agents that inhibit PFKFB4 is to determine whether the kinase or phosphatase domain of this bifunctional enzyme are active in neoplastic cells. Whereas the kinase activity will produce F2,6BP and increase flux down the glycolytic pathway producing ATP, the phosphatase activity will decrease F2,6BP, reduce PFK-1 activity and increase the oxidative pentose shunt activity and NADPH via the availability of glucose 6-phosphate which is in equilibrium with fructose 6-phosphate (F6P) through glucose 6-phosphate isomerase. Given that the intracellular concentration of the substrate of the kinase domain (F6P) is >10,000 fold the substrate of the phosphatase domain (F2,6BP) in transformed cells (*e.g.* MCF-7 cells: F6P, 50±24 nmol/mg protein [16]; F2,6BP, 2.97±.21 pmol/mg protein [17]), we predicted that the kinase activity of PFKFB4 would be the dominant function of this enzyme in transformed cells.

In the present study, we find that PFKFB4 functions primarily to produce F2,6BP, thus stimulating glycolytic flux to lactate and the Kreb's cycle in order to generate ATP and anabolic precursors. Accordingly, the synthesis of small molecule inhibitors of the PFKFB4 kinase domain is anticipated to enable the development of novel agents for the treatment of cancer.

## RESULTS

### High Expression of PFKFB4 in Several Normal Tissues and in Lung Adenocarcinomas

**Figure 1:**
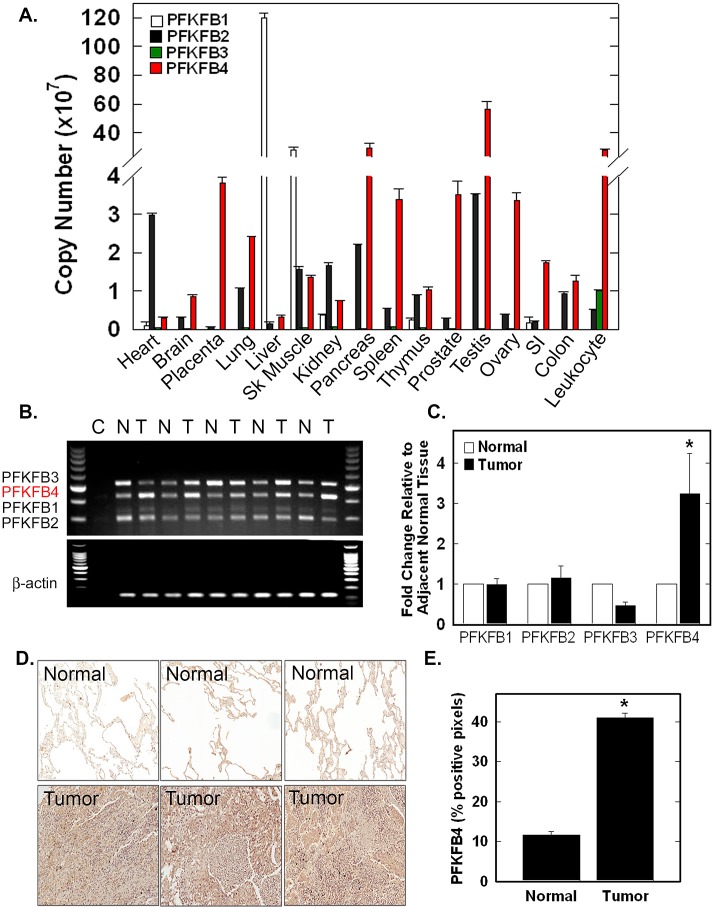
PFKFB4 Is Expressed in Multiple Normal Tissues and Over-Expressed in Human Lung Adenocarcinomas PFKFB1-4 mRNA expression in multiple normal tissues was assessed by real-time RT-PCR and expressed by copy number (A). Matched normal (N) and lung adenocarcinoma (T) tissue were analyzed by multiplex RT-PCR (B) and by real-time RT-PCR expressed as fold change relative to adjacent normal tissues (C). Immunohistochemical staining of lung adenocarcinomas and normal lung was conducted on patients’ biopsies, three representative sections are shown (D) and positive pixels were quantitated (E). Data are expressed as the mean ± SD of three experiments. *p value < 0.001 compared to normal tissue controls.

We first conducted a simultaneous analysis of PFKFB1-4 mRNA expression in normal human tissues using real-time RT-PCR for each family member. We observed high expression of: PFKFB1 in liver and skeletal muscle; PFKFB2 in heart, lung, skeletal muscle, kidney, pancreas and testes; and PFKFB4 in placenta, lung, skeletal muscle, pancreas, spleen, prostate, testes, ovary, colon and leukocytes (Fig. 1A). PFKFB3, which has been characterized as being inducible [18] and ubiquitous [19], was expressed at very low levels in all tissues with the exception of leukocytes (Fig 1A). We then used multiplex RT-PCR and real-time RT-PCR to assess PFKFB1-4 mRNA expression in normal lung tissues and adjacent lung adenocarcinomas from five patients. Only PFKFB4 mRNA was over-expressed in each of these patients’ tumors (Fig. 1B, C). Further examination of a larger cohort of lung adenocarcinomas and adjacent normal tissues from 18 patients by immunohistochemical analyses revealed high PFKFB4 protein expression relative to normal lung alveoli (Fig. 1D, E).

### The Kinase:Phosphatase Ratio of PFKFB4 is 4.3:1 and Selective Inhibition of PFKFB4 Expression Reduces F2,6BP in Multiple Cancer Cell Lines

**Figure 2:**
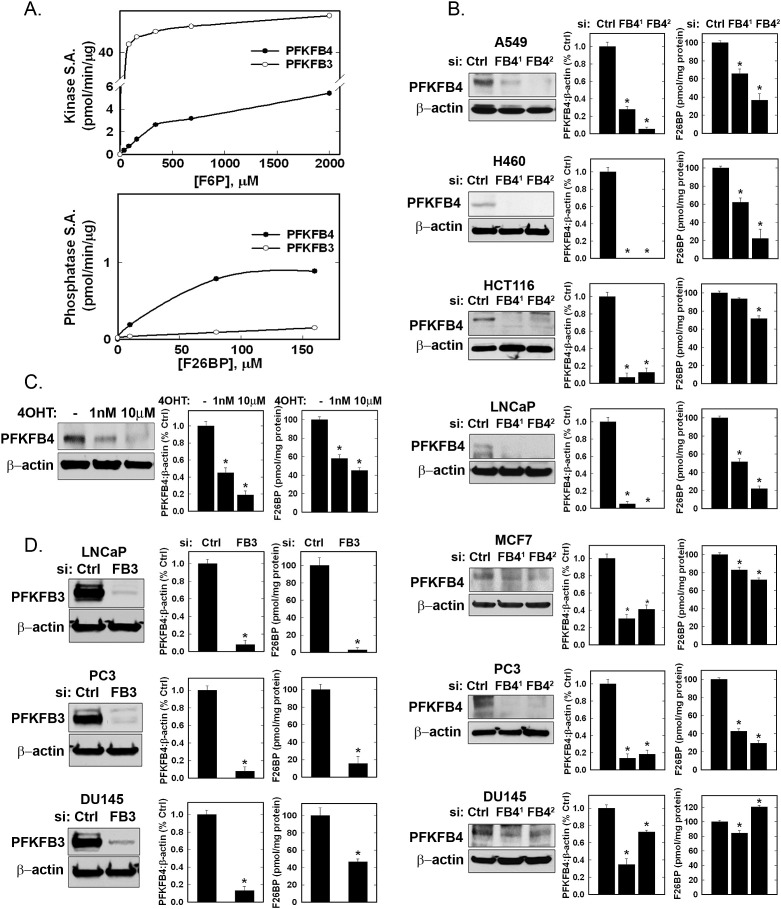
Recombinant PFKFB3 and PFKFB4 Kinase and Phosphatase Activities and Effects of PFKFB3 and PFKFB4 Inhibition on Intracellular F2,6BP in Cancer Cells Human PFKFB3 and PFKFB4 were expressed and relative kinase and phosphatase specific activities (S.A.) were determined (A). The indicated cancer cell lines were transfected with control siRNA (Ctrl) or two PFKFB4 siRNAs (FB4^1^ and FB4^2^) and, after 48 hours, protein expression examined by Western blot and quantitated by densitometry and intracellular F2,6BP was measured (B). Large T antigen-immortalized, tamoxifen (4OHT)-inducible *Pfkfb4*^−/−^ lung fibroblasts were exposed to 1 nM and 10 μM 4OHT (ethanol used as vehicle control) and analyzed for protein expression and F2,6BP after 48 hours (C). The indicated cancer cell lines were transfected with an established PFKFB3 siRNA and analyzed for protein expression and F2,6BP 48 hours later (D). Data are expressed as the mean ± SD of three experiments. * *p* value <0.001 compared to vehicle or control siRNA.

We expressed and purified recombinant human PFKFB4 and compared the kinase and phosphatase activities to those of the inducible PFKFB3 recombinant protein. Given the recent reports that PFKFB4 functions as a dominant bisphosphatase in cancer cells [15], we were surprised to find that recombinant human PFKFB4 had significant kinase activity that was ~15% that of the PFKFB3 family member whose kinase domain has previously been established to set the intracellular concentration of F2,6BP in multiple cell lines (PFKFB4 kinase V_max_ = 5.06±0.2 mU/mg, PFKFB3 kinase V_max_= 38.4 ±2.3 mU/mg) (Fig. 2A) [18, 20, 21]. In contrast, both PFKFB4 and PFKFB3 were found to have relatively low phosphatase activities (PFKFB4 bisphosphatase V_max_ = 1.17±0.02 mU/mg; PFKFB3 bisphosphatase V_max_ = 0.47±0.035 mU/mg) and the kinase:bisphosphatase ratios for PFKFB4 and PFKFB3 were calculated to be 4.3:1 and 81.7:1, respectively (Fig. 2A). Since the concentration of the kinase substrate, F6P, is so much higher than that of the bisphosphatase substrate, F2,6BP (*e.g.* H460 cell [F6P] = 12518±1104 pmol/mg protein; [F2,6BP] = 4.47±.21 pmol/mg protein) and because the kinase:phosphatase is 4.3:1, we predicted that selective inhibition of PFKFB4 would preferentially inhibit the kinase function of this bifunctional enzyme and thus result in a reduction in F2,6BP. We found that this did indeed occur in six out of seven transformed cancer cell lines derived from lung, colon, prostate and breast that were each transfected with two separate PFKFB4-specific siRNAs (Fig. 2B). Importantly, genomic deletion of *Pfkfb4* in LT-immortalized fibroblasts also caused a marked reduction in F2,6BP** (Fig. 2C). Since PFKFB3, unlike PFKFB4, has been established to function as a kinase in transformed cells, we next examined the effect of PFKFB3 siRNA transfection on three transformed cell lines and similarly observed a marked reduction in the steady-state concentration of F2,6BP (Fig. 2D).

### Selective Inhibition of PFKFB4 Expression Reduces Glycolysis and ATP in Multiple Cancer Cell Lines

**Figure 3:**
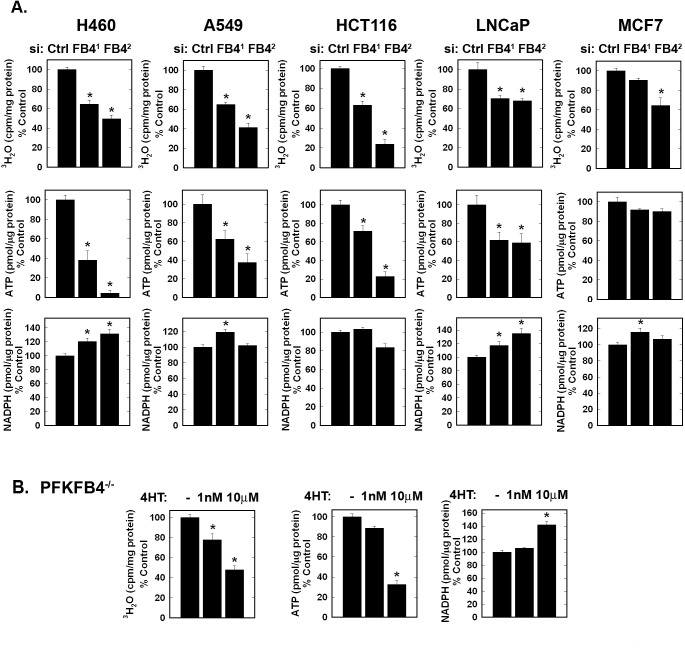
Effects of PFKFB4 Inhibition on Cancer Cell Glycolysis, ATP and NADPH The indicated cell lines were transfected with control siRNA (Ctrl) or two PFKFB4 siRNAs (FB4^1^ and FB4^2^) and glycolysis (^3^H_2_O production from [5-^3^H]glucose), intracellular ATP and NADPH were measured (A). Large T antigen-immortalized, tamoxifen (4OHT)-inducible *Pfkfb4*^−/−^ lung fibroblasts were exposed to 1 nM and 10 μM 4OHT and analyzed for glycolysis, ATP and NADPH 48 hours later (B). Data are expressed as the mean ± SD of three experiments. * *p* value <0.01 compared to vehicle or control siRNA.

We examined the effect of the two PFKFB4 siRNA molecules and genomic deletion of *Pfkfb4* on glycolysis (measured by the production of ^3^H_2_O from [5-^3^H]glucose), ATP and NADPH. We predicted that if the kinase function of PFKFB4 is dominant, then PFKFB4 inhibition would suppress (rather than increase) glycolysis and decrease the concentration of ATP which is produced downstream of PFK-1. We found that both glycolysis and ATP were reduced in all five cell lines examined although the ATP depletion in MCF-7 cells was not statistically significant (Fig. 3A). Although we observed variable effects on NADPH, we did find that PFKFB4 inhibition increased NADPH in H460, A549, LNCaP and MCF-7 cells, presumably as a result of increased availability of F6P for the activity of glucose 6-phosphate isomerase and the oxidative pentose shunt (Fig. 3A). Importantly, we found that *Pfkfb4* genomic deletion reduced glycolysis and ATP and increased NADPH, all findings consistent with a requirement of PFKFB4 for the activation of PFK-1 (Fig. 3B).

### Over-Expression of PFKFB4 Increases F2,6BP, Glycolysis and ATP and reduces NADPH

**Figure 4:**
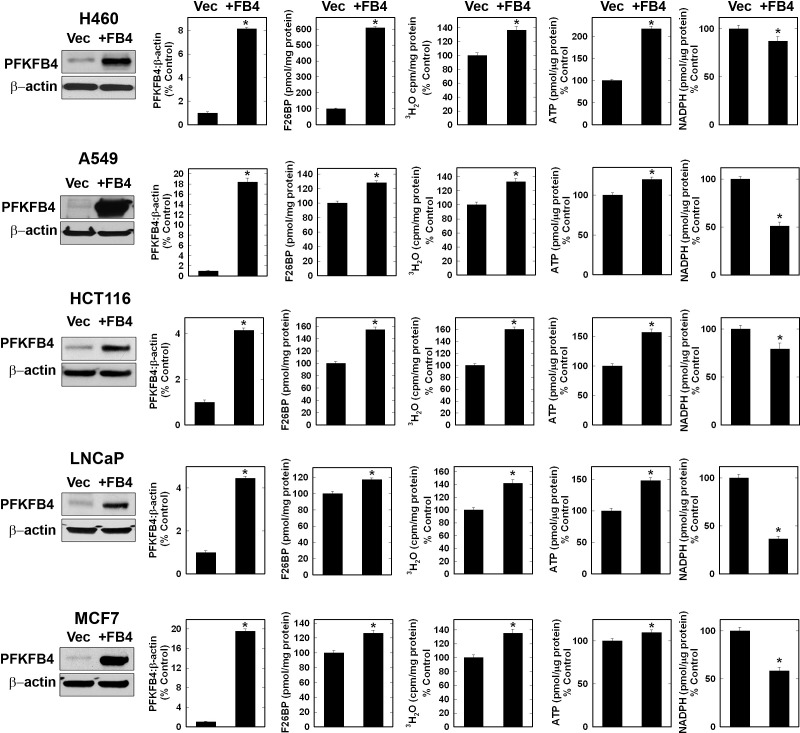
Over-Expression of PFKFB4 in Cancer Cells Increases F2,6BP, Glycolysis and ATP but Decreases NADPH The indicated cancer cell lines were transfected with either empty pCMV-XL4 (Vec) or pCMV-XL4 containing full-length PFKFB4 (+FB4) and analyzed 48 hours later by Western blot and densitometry, and for F2,6BP, glycolysis, ATP and NADPH. Data are expressed as the mean ± SD of three experiments. * *p* value < 0.01 compared to empty pCMV-XL4 vector.

Given that the kinase activity of PFKFB4 is 4.3-fold the phosphatase activity and that PFKFB4 siRNA transfection and genomic deletion both reduced F2,6BP, we predicted that over-expression would have the opposite effect on F2,6BP and subsequent metabolic events. We found that PFKFB4 over-expression increased F2,6BP, glycolysis and ATP, and decreased NADPH in each of the examined transformed cell lines (Fig. 4). We also examined the effect of addition of 5 mM pyruvate on the depletion of ATP caused by PFKFB4 siRNA in H460 cells and found that pyruvate partially reversed the ATP depletion, further supporting a role for PFKFB4 in increasing the glycolytic production of ATP (ATP, pmoles/μg protein: Ctrl siRNA, 39.9±0.2; PFKFB4 siRNA 9.94±0.5; Ctrl siRNA + Pyruvate, 40.4±0.13; PFKFB4 siRNA + pyruvate, 33.1± 1.1; *p* value < 0.01).

### Inhibition of PFKFB4 Suppresses H460 Lung Adenocarcinoma F2,6BP, Glucose Uptake and Growth *In Vivo*

**Figure 5:**
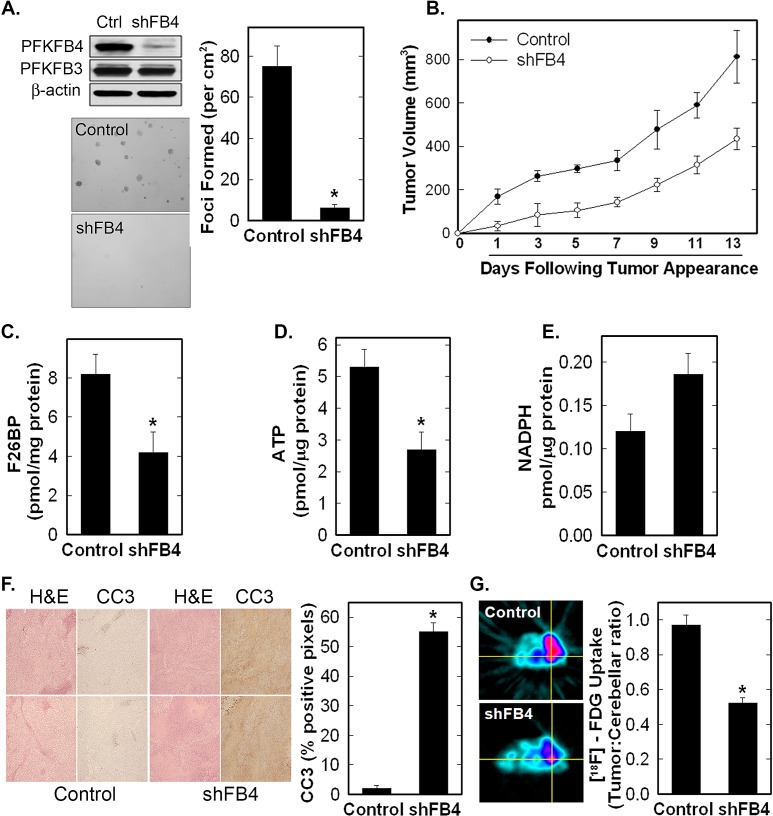
Stable shRNA Knock-Down of PFKFB4 Reduces Tumor Growth, Glucose Uptake and F2,6BP, and Increases Apoptosis H460 cells were stably transduced with PFKFB4 (shFB4) or control (Control, Ctrl) shRNA and assessed for PFKFB4 and PFKFB3 protein expression by Western blot analysis (A), soft agar colony formation (A), tumor growth in athymic mice (B). F2,6BP concentration (C), ATP (D), and NADPH (E) were measured in tumors following resection. Data are expressed as the mean ± SD of three experiments. Tumor sections were examined for apoptosis by cleaved caspase 3 (CC3) immunohistochemistry, with representative images on the left and positive pixels quantified on the right (F). The data is depicted as % positive pixels/total pixels ± SD. Tumors in mice were examined for ^18^F-FDG uptake *in vivo* by micro-PET imaging. Regions of interest in the tumors and cerebellum were quantified in quadruplicate (right) and representative transverse cuts are shown on the left (G). *p value < 0.001 compared to control shRNA.

In order to determine if PFKFB4 functions to synthesize F2,6BP *in vivo,* we stably transfected H460 cells with PFKFB4 shRNA and confirmed reduced PFKFB4 protein expression *in vitro* (Fig 5A). We found that stable PFKFB4 inhibition reduced anchorage-independent growth as soft agar colonies and as tumors (Fig. 5A, B). We then examined resected tumors after two weeks of growth and observed a marked reduction in tumor F2,6BP and ATP and an increase in apoptotic cells in the tumors by immunohistochemistry for cleaved caspase 3 (Fig 5C, D, F) We also found a small increase in NADPH in the PFKFB4 shRNA tumors in comparison with control tumors which was not statistically significant (Fig 5E). In addition, we examined uptake of [^18^F]-FDG in PFKFB4 shRNA and control tumors by PET scan and observed reduced tumor [^18^F]-FDG uptake in the PFKFB4 shRNA tumors (Fig. 5G) as has also been described as a result of PFKFB3 inhibition. Taken together, these *in vivo* studies provide strong evidence that PFKFB4 supports tumor growth by functioning as a kinase to synthesize F2,6BP.

### PFKFB4 mRNA and Protein Expression Are Increased by Hypoxia and Required for Hypoxia-Induced F2,6BP, Glucose Uptake and Glycolysis

**Figure 6:**
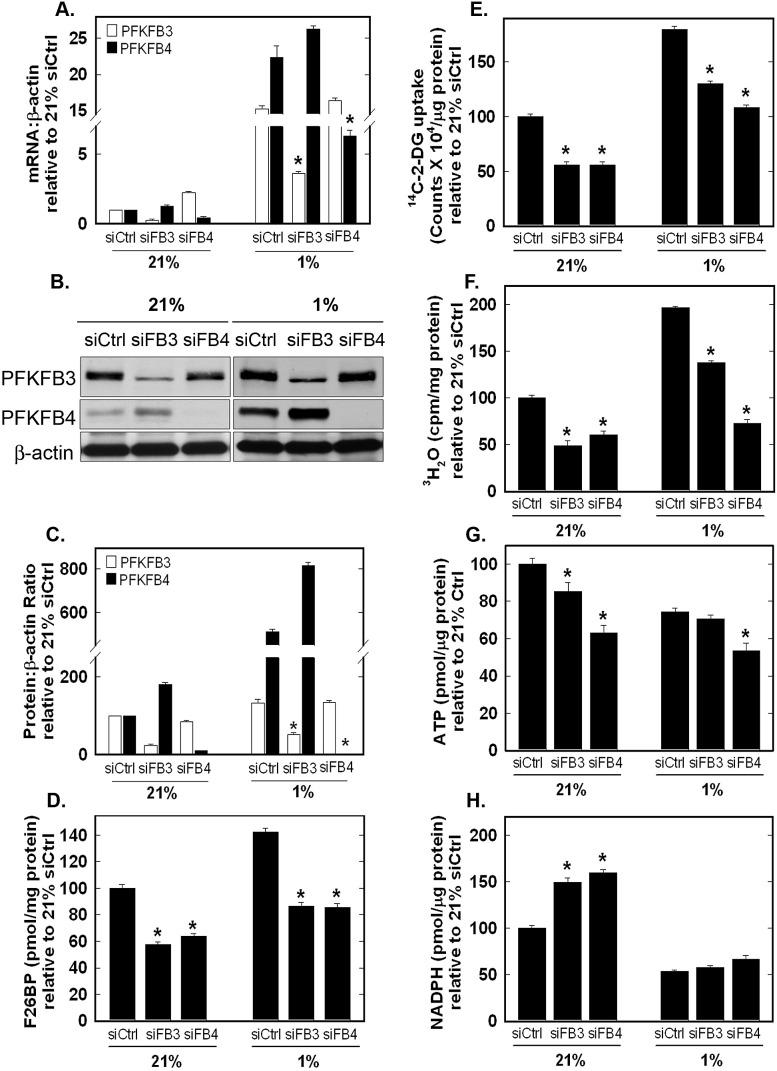
Role of PFKFB3 and PFKFB4 in Hypoxia-Induced F2,6BP production, Glucose Uptake, Glycolysis, ATP and NADPH H460 cells were transfected with control siRNA (siCtrl), PFKFB3 siRNA (siFB3) or PFKFB4 siRNA (siFB4), cultured in 21% oxygen or 1% oxygen and examined for PFKFB3 and PFKFB4 mRNA expression (A) and protein expression by Western blot analysis and densitometry (B and C), F2,6BP (D), ^14^C-2-DG uptake (E), glycolysis (^3^H_2_O production from [5-^3^H]glucose) (F), intracellular ATP (G) and NADPH (H). Data are expressed as the mean ± SD of three experiments. * *p* value < 0.01 compared to control siRNA.

Both PFKFB3 and PFKFB4 mRNA previously have been found to be induced by hypoxia [10, 12, 13, 22-24] and we confirmed these past observations in H460 cells (Fig. 6A). In order to compare the functional relevance of PFKFB3 and PFKFB4 under hypoxic conditions, we transfected H460 cells with PFKFB4 or PFKFB3 siRNA under 21% and 1% oxygen. We observed a reduction in the hypoxic induction of both enzyme mRNAs as a result of the siRNA molecules (Fig. 6A). Hypoxia strongly induced PFKFB4 protein expression, as has been observed in other cell lines (Fig. 6A-C). Interestingly, we found a far lower induction of PFKFB3 protein which may already be maximally expressed as a result of the multitude of genetic alterations that increase PFKFB3 protein expression such as *PTEN* loss and Ras activation (*e.g.* H460 cells express *KRAS^Q61H^*) [20, 25, 26]. Importantly, transfection with either PFKFB4 or PFKFB3 siRNA caused an attenuation of F2,6BP, glucose uptake and glycolysis stimulated by hypoxia (Fig. 6D-F). The concentrations of both ATP and NADPH were decreased under hypoxia and PFKFB4 siRNA exacerbated the ATP decrease, indicating that PFKFB4 may be essential for the glycolytic response to hypoxia in these cells (Fig. 6G, H).

### PFKFB3 or PFKFB4 Inhibition in H460 Cells Reduces Glycolytic Flux to Lactate and Glutamate

**Figure 7:**
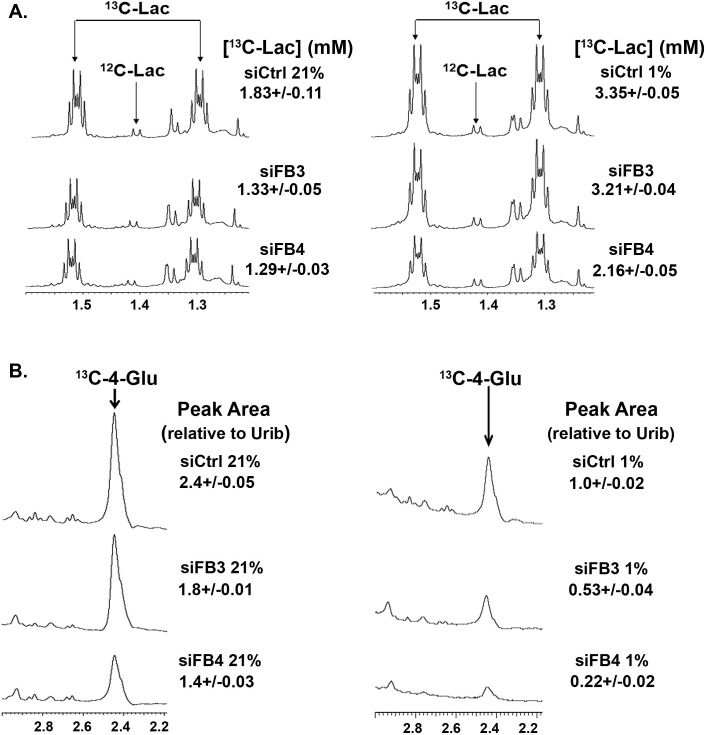
Role of PFKFB3 and PFKFB4 in the Conversion of ^13^C-Glucose into ^13^C-Lactate and ^13^C-4-Glutamate H460 cells were transfected with control siRNA (siCtrl), PFKFB3 siRNA (siFB3) or PFKFB4 siRNA (siFB4), cultured in 21% oxygen or 1% oxygen, pulsed with uniformly-labeled 1^3^C-glucose for hours and extracted for NMR analyses of ^13^C-lactate and ^13^C-4-glutamate. (A) 1D NMR spectra of media extracts showing the region corresponding to the methyl groups of lactate and essential amino acids. Satellite peaks were integrated and normalized to internal DSS concentration to give the concentrations noted. Under these conditions, nearly all of the lactate derives from glucose. (B) 1D ^1^H{^13^C} HSQC spectra of cellular extracts showing selective enrichment at the C4 atom of glutamate (Glu) under normoxia, (21 % O_2_, left) and under hypoxia (1% O_2_, right). The C4 position of Glu becomes enriched by addition of glucose-derived acetate to oxaloacetate. The resulting ^13^C2 citrate is converted by the TCA cycle to 2-oxoglutarate and transaminated to Glu, showing that the TCA cycle remains active in these cells, but is greatly reduced under 1% oxygen and by PFKFB3 and PFKFB4 knockdown. Values on the right denote ratios of peak areas normalized to uridine ribose (Urib) H1’, which correspond to relative amounts of glucose-derived ^13^C. Numbers expressed are the mean ± SD of three experiments.

Given that both PFKFB3 and PFKFB4 siRNA suppressed hypoxia-induced F2,6BP, we suspected that inhibition of PFKFB3 or PFKFB4 would result in reduced glycolytic flux to lactate and into the TCA cycle product, glutamate, under hypoxic conditions. Whereas we found that both PFKFB3 and PFKFB4 siRNAs caused a reduction in the conversion of ^13^C-glucose to lactate and glutamate as assessed by NMR, the PFKFB4 siRNA caused a far greater reduction in the conversion to both products under both normoxic and hypoxic conditions (Fig. 7A, B).

### PFKFB3 or PFKFB4 Inhibition in H460 Cells Increases Apoptosis Under Normoxic and Hypoxic Conditions

**Figure 8:**
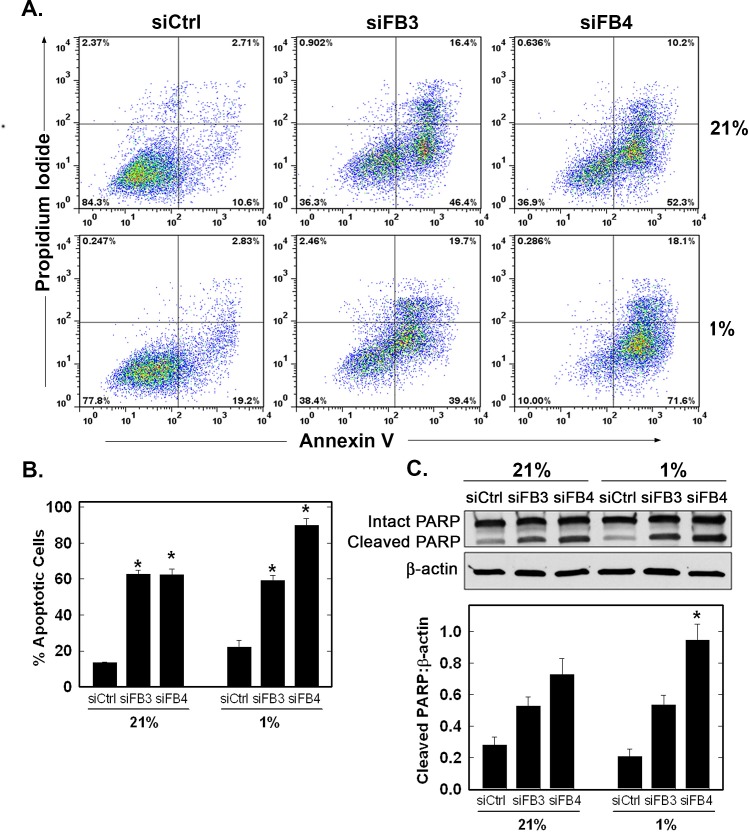
Effect of PFKFB3 or PFKFB4 siRNA Transfection on Apoptosis H460 cells were transfected with control siRNA (siCtrl), PFKFB3 siRNA (siFB3) or PFKFB4 siRNA (siFB4), cultured in 21% oxygen or 1% oxygen for 48 hours, and analysed for apoptosis by flow cytometry of propidium iodide and annexin V positive cells (A and B, PI^+^ + PI/Ann V^+^ cells shown as % apoptotic cells) and by cleaved PARP by Western blot analysis and densitometry (C). Data are expressed as the mean ± SD of three experiments. * *p* value < 0.005 compared to control siRNA.

Maintenance of intracellular ATP is required to prevent apoptosis and we postulated that PFKFB3 or PFKFB4 inhibition would increase apoptosis. We observed a marked increase in apoptotic PI^+^/Annexin V^+^ cells and cleaved PARP with inhibition of either family member under both normoxia and hypoxia but observed the greatest number of apoptotic cells as a result of the combination of hypoxia and PFKFB4 inhibition (Fig. 8A-C).

### PFKFB4 Expression Correlates With Hypoxia in Human Lung Adenocarcinoma Xenografts

**Figure 9:**
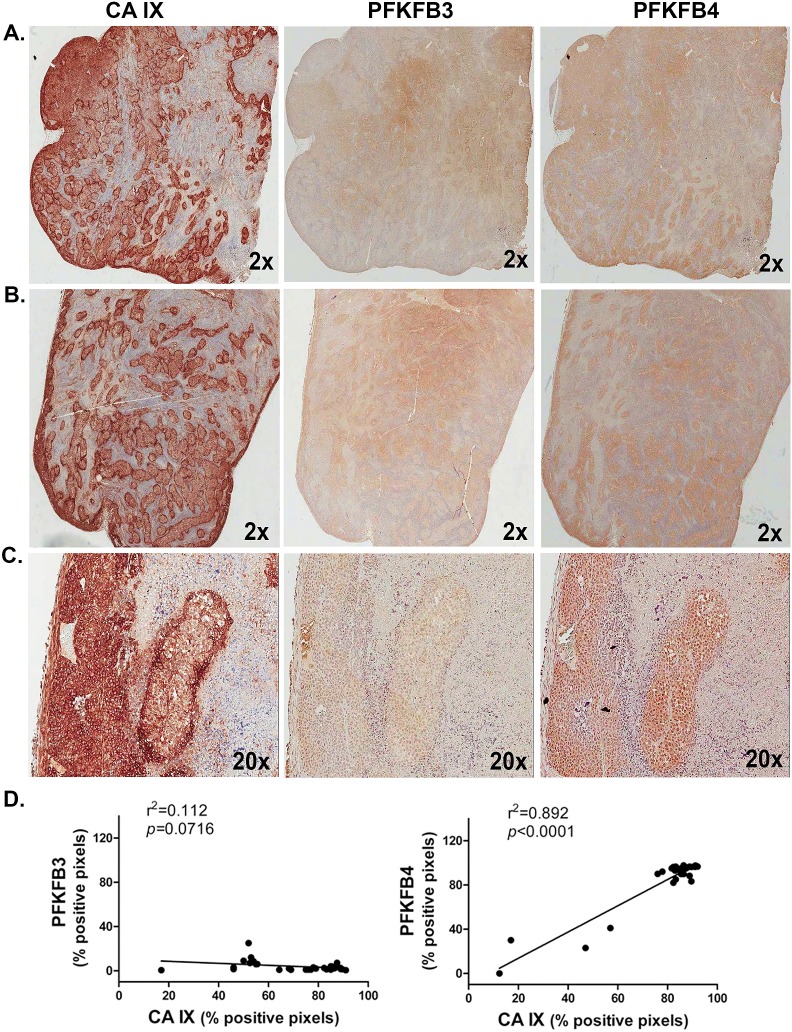
PFKFB4 Expression Correlates with Hypoxia In Human Lung Adenocarcinoma Xenografts Ten human H460 lung adenocarcinoma xenografts were analyzed by immunohistochemistry for carbonic anhydrase (CA) IX, PFKFB3 and PFKFB4. Representative adjacent sections from two tumors are provided (A,B). 20X magnification of portion of tumor in (B) shown (C). Positive pixels were enumerated in a minimum of 5 fields per tumor section followed by linear regression analysis (D). r^2^ and *p* values are provided.

We examined serial sections of human H460 lung adenocarcinoma xenograft tumors resected from athymic mice for expression of PFKFB4 and PFKFB3 and correlated the expression levels of these proteins using carbonic anhydrase IX, a potent transcriptional target of HIF-1α, as a hypoxia marker. We observed a statistically significant correlation between PFKFB4 and carbonic anhydrase IX expression but observed no correlation between PFKFB3 and carbonic anhydrase IX (Figure 9A-D).

## DISCUSSION

The main objective of this study was to determine which of the two enzymatic domains of PFKFB4 is active in transformed cells. Using multiple cell types and approaches, we found that: (i) recombinant human PFKFB4 has far more kinase activity than phosphatase activity (kinase:phosphatase ratio = 4.3:1); (ii) selective inhibition of PFKFB4 using two unique siRNA molecules reduces the intracellular F2,6BP in six of seven cancer cell lines indicating that endogenous PFKFB4 synthesizes F2,6BP in these cancer cells; (iii) over-expression of PFKFB4 increases the F2,6BP in all examined cancer cell lines; and (iv) genomic deletion of the *Pfkfb4* gene reduces F2,6BP in LT-immortalized fibroblasts. Taken together, these *in vitro* data provide overwhelming evidence that the main biochemical function of PFKFB4 is to synthesize F2,6BP. We also found that PFKFB4 siRNA reduces glycolysis and ATP and increases the steady-state concentration of NADPH *in vitro*, all expected metabolic effects of reduced F2,6BP. Additionally, we observed that PFKFB4 shRNA suppressed the glucose uptake, F2,6BP, ATP and growth of H460 xenograft tumors in mice, thus providing further evidence that the main function of PFKFB4 *in vivo* is to synthesize F2,6BP and thus activate PFK-1 and glycolysis. The rescue of transformed cells from PFKFB4 inhibition with the addition of pyruvate, which provides substrate for mitochondrial ATP production, provides further support that PFKFB4 is predominantly functioning to stimulate ATP production. To summarize, these data indicate that the kinase activity and, thus, the capacity to produce F2,6BP, serves as the dominant function of PFKFB4 in cancer cells.

Since the majority of human cancers display elevated glucose uptake and several targeted agents such as the BRAF inhibitor, vemurafenib, and the estrogen antagonist, fulvestrant, acutely suppress glucose metabolism in order to inhibit tumor growth [17, 27], we believe that PFKFB3 and PFKFB4 *kinase* inhibitors may prove effective for the treatment of cancer. Importantly, a novel family of related small molecule antagonists of the kinase domain of PFKFB3 (*i.e.* 3PO, PFK15 and PFK158) have been found to reduce glycolytic flux, ATP and cancer cell viability [17, 21, 28]. PFK158 is now being tested in a phase I clinical trial of advanced cancer patients (clinicaltrials.gov #NCT02044861) and recently has been found to synergistically interact with targeted cancer agents [17]. Given that PFKFB3 and PFKFB4 are co-expressed in several cancer cell lines and have the redundant function of synthesizing F2,6BP, we postulate that selective inhibition of a single family member may result in compensation by a co-expressed PFKFB family member, and that combinations of PFKFB3 and PFKFB4 inhibitors may be necessary in order to fully suppress tumor glucose metabolism and growth. Future studies will be directed at creating double *Pfkfb3* and *Pfkfb4* knockout mice in order to conduct *in vivo* studies of the roles of these two seemingly redundant enzymes in regulating the glucose metabolism and growth of tumors.

Although this is the first comprehensive analysis of the relative importance of the kinase activity of PFKFB4 in transformed cells to include a measurement of human recombinant protein activity and a combination of siRNA knock-down, plasmid-based over-expression and genomic deletion experiments in multiple cancer cell types, two prior studies previously have supported the conclusion that the kinase domain of PFKFB4 was essential for cancer cell proliferation. A PFKFB4 siRNA was found to reduce F2,6BP and glycolytic flux to lactate in A549 lung adenocarcinoma cells (U.S. Patent #8,283,332) and two other PFKFB4 siRNAs were independently observed to reduce lactate secretion and the intracellular ATP in malignant glioma cells [14]. However, a third study found that PFKFB4 siRNA increased the F2,6BP concentration of three prostate cancer cell lines [15] including the LNCaP and PC3 cell lines (using one of the two siRNAs examined in the current study). Although it is difficult to reconcile the findings from this single discordant report with those of the other two studies as well as the present study, we believe that the combined use of recombinant protein analyses, cell lines derived from several types of cancer, two methods to inhibit PFKFB4 (siRNA and genomic deletion) and PFKFB4 over-expression experiments provide comprehensive and compelling data demonstrating that the kinase activity of PFKFB4 dictates the F2,6BP concentration in transformed cells. Whereas we look forward to continued independent examinations of the biochemical functions of PFKFB4 in transformed cells, our current data support an essential role of the PFKFB4 kinase activity for cancer cell survival as well as the development of small molecule antagonists that target the kinase domain of PFKFB4.

Although the differences in metabolic functions of PFKFB3 and PFKFB4 are poorly understood, we observed a more robust induction of PFKFB4 protein relative upon exposure of H460 cells to 1% oxygen and significant correlation between hypoxia and PFKFB4 expression in lung adenocarcinoma tissues. Additionally, we detected a greater reduction in glycolytic flux to glutamate and lactate caused by PFKFB4 siRNA relative to PFKFB3 siRNA transfection. Given these results, we suspect that PFKFB4 may serve as a unique regulator of the glycolytic response to hypoxia. This hypothesis is supported by the observation that the H460 cells underwent increased hypoxia-associated apoptosis when subjected to PFKFB4 siRNA transfection but not when subjected to PFKFB3 siRNA transfection. These data suggest that tumors that are especially affected by poor vasculature and hypoxia may be most responsive to PFKFB4 inhibition.

## CONCLUSIONS

In conclusion, our data indicate that the PFKFB4 family member functions predominantly to synthesize F2,6BP which, in turn, is required for glycolytic flux through PFK-1 and subsequent ATP production. This conclusion is consistent with the finding that H460 cells are exquisitely sensitive to PFKFB4 inhibition under hypoxic conditions when the efficiency of ATP production by the mitochondria is compromised and the reliance on glycolysis to produce ATP is greatest. With the key intramolecular target of PFKFB4 now established, we believe that PFKFB4 inhibitors can be rationally designed and that these agents may prove most useful when combined with PFKFB3 inhibitors in order to circumvent potential compensation between these two PFKFB family members.

## METHODS

### Cell lines and cell culture

A549 and H460 NSCLC, MCF7 breast adenocarcinoma, LNCaP, PC3 and DU145 prostatic adenocarcinoma and HCT116 colon adenocarcinoma cell lines were obtained from ATCC (Manassas, VA). PFKFB4^−/−^ ear pinna fibroblasts were isolated from TamCre/loxP/PFKFB4^−/−^ mice and immortalized by transduction with REBNA/IRES retrovirus expressing SV40 large T antigen as described previously [20]. Cell lines were grown in DMEM (A549, LNCaP, PC3, DU145 and PFKFB4^−/−^), RPMI 1640 (H460), McCoy's 5A media (HCT116) or improved MEM (MCF7) (all from Invitrogen, Grand Island, NY) containing 10% fetal calf serum (FCS, Hyclone, Logan, UT) at 37ºC in 5% CO2. In some experiments, 4-hydroxytamoxifen (4OHT, 4HT, Sigma -Aldrich, St. Louis, MO) was added to PFKFB4^−/−^ fibroblasts at indicated concentrations.

### Transfections

For siRNA experiments, cells growing in 6-well plates were transfected with control siRNA (Stealth Negative Control Medium GC Duplex, Invitrogen), PFKFB3 siRNA (FB3; HSS107860, Invitrogen) or PFKFB4 siRNA (FB4^1^, FB4; HSS107863, Invitrogen and FB4^2^; siGENOME human PFKFB4 5210 #2, Dharmacon) using Lipofectamine RNAiMax (Invitrogen, Carlsbad, CA), and harvested 48 hours after transfection. For hypoxia experiments, cells were transfected with siRNA and, after 24 hours, were placed in a hypoxia chamber (Billups-Rothenburg, Del Mar, CA) purged with 1% oxygen for 24 hours. For short hairpin RNA (shRNA) experiments, sense and antisense DNA oligonucleotides for PFKFB4 were designed with a hairpin against the target sequences 5′-cacttgtatggtcctgt-3′ and 5′-ggagagcgaccatcttt-3′ were produced by IDT (Coralville, IA). The oligonucleotides were annealed and ligated into pSUPER.neo vector (OligoEngine, Seattle, WA) following manufacturer's instructions. H460 cells were transfected with an shRNA-expressing plasmid targeted against PFKFB4 (shFB4) or a scrambled shRNA expressing plasmid (Control) using Lipofectamine 2000 (Invitrogen, Carlsbad, CA) and clones selected with G418 (500 μg/ml). For overexpression experiments, cells were transfected with pCMV-XL4 (vector) or pCMV-XL4 containing full-length PFKFB4 (Origene, Rockville, MD) using Lipofectamine 2000 and harvested after 48 hours.

### PCR analyses

Multiplex mRNA primers were custom synthesized (IDT) against human PFKFB1-4 as described previously [20]. cDNA from normal human tissues and matched tumor and adjacent normal tissues (Clontech, Mountain View, CA) were analyzed using these primers and standard PCR conditions. PFKFB1-4 mRNA expression was determined using real-time RT-PCR with TaqMan probes for human PFKFB1-4 and β-actin (Applied Biosystems, Foster City, CA) in triplicate in 96-well optical plates (MicroAMP®, Applied Biosystems). Analysis of results and fold differences between samples were determined using StepOne software (version2.1) (Applied Biosystems) and calculated from the ΔΔCT values with the formula (2^−ΔΔCT^). The data are represented as the mean ± SD from triplicate measurements from three independent experiments. For calculation of copy number, the molecular weight was determined for the double stranded DNA sequences of the 4 PFKFB isoforms. The OD of the DNA (in gm/μL) was divided by the molecular weight of the product (result in moles/μL) and then this number was multiplied by Avogadro's number (6.022 × 10^23^ molecules/mole). The resulting number of DNA molecules per μL was used to generate a standard curve from which copy numbers were calculated. Statistical significance was assessed by the two-sample t test (independent variable).

### Protein extraction and Western blotting

Cells were harvested, washed X1 in PBS and lysed in 1X lysis buffer (Pierce Biotechnology, Rockford, IL) containing protease inhibitors. Protein samples were resolved on 4-20% SDS-PAGE gels (BioRad, Hercules, CA) and transferred to PVDF membranes (BioRad). After blocking in TBS containing 0.1% Tween 20 (TBS-T) and 5% milk, membranes were probed with antibodies to PFKFB3 (Proteintech, Chicago, IL), PFKFB4 (Abcam, Cambridge, MA) or β-actin (Sigma, St. Louis, MO). Secondary antibodies used were HRP-conjugated goat anti-rabbit or anti-mouse (1:5000, Pierce Biotechnology). Scanned images were quantified by densitometric analyses using Image J software (http://rsb.info.nih.gov/ij/). Values obtained were normalized to β-actin and expressed in densitometric units as a percentage of control. The data represented are the mean ± SD from triplicate measurements from three independent experiments. Statistical significance was assessed by the two-sample t test (independent variable).

### Kinase and Bisphosphatase assays

Fructose-6-phosphate kinase and fructose-2,6-bisphosphatase activities of human recombinant PFKFB3 and PFKFB4 were assayed using previously described methods wherein one unit of activity was defined as the amount of enzyme that catalyzes the formation of 1 μmol of F2,6BP per min. [7, 8]. Aliquots of the reaction mixture were removed at intervals, added to 0.1N NaOH and then neutralized to a pH of 7.2 and assayed for F2,6BP as described below.

### F2,6BP measurements

Cells were harvested, washed twice with PBS, lysed in NaOH/Tris acetate by heating at 80°C for 5 min. Lysates were neutralized to pH 7.2 with ice-cold acetic acid and HEPES. F2,6BP content was measured using a coupled enzyme reaction following the method of Van Schaftingen *et al* [29]. The F2,6BP concentration was normalized to total cellular protein measured by the bicinchoninic acid assay (BCA, Thermo Scientific, Rockford, IL). All data are expressed as the mean ± SD of three experiments. Statistical significance was assessed by the two-sample t test (independent variable).

### Glycolysis assay

Cells growing in 6-well plates were incubated in 500 μl of complete medium containing 1 μCi of 5-[^3^H] glucose per well for 60 min in 5% CO_2_ at 37°C. The medium was then collected and centrifuged for 5 min at 8000 rpm to pellet any suspended cells. To separate the ^3^H_2_O formed via glycolysis from the 5-[^3^H]glucose added to the medium, an evaporation technique in a sealed system was utilized. Briefly, 150 μl aliquots of medium were added to open tubes that were placed upright inside scintillation vials containing 1 ml of H_2_O. The scintillation vials were sealed, and the ^3^H_2_O produced by glycolysis through enolase and released to the medium was allowed to equilibrate with the H_2_O in the outer vial for 48 h at 37°C. The amounts of ^3^H_2_O that had diffused into the surrounding H_2_O was measured on a Tri-Carb 2910 liquid scintillation analyzer (Perkin Elmer, Boston, MA) and compared with ^3^H_2_O and 5-[3H]glucose standards. Protein concentration was determined using the BCA assay and counts were normalized to protein concentration. All data are expressed as the mean ± SD of three experiments. Statistical significance was assessed by the two-sample t test (independent variable).

### 2-[1-^14^C]-Deoxy-D-Glucose Uptake

Cells were placed in glucose-free media for 30 minutes, 2-[1-^14^C]-deoxy-D-glucose (0.25 μCi/mL; Perkin Elmer) was added for an additional 60 min and the cells then were washed thrice with ice-cold glucose-free media. Cell lysates were collected in 500 μL of 0.1% SDS, and scintillation counts (counts/min) were measured on 400 μL of the lysate. Counts were normalized to protein concentration measured by the BCA assay and data are represented as mean ± SD from triplicate measurements from three independent experiments. Statistical significance was assessed by the two-sample t test (independent variable).

### ATP Measurements

Cell pellets were lysed using Passive Lysis buffer (1X, Molecular Probes, Invitrogen). Lysates were flash frozen in liquid nitrogen and thawed (to 37ºC) once to accomplish complete lysis and then centrifuged at 4ºC for 30 seconds to clear the lysates. Intracellular ATP levels were determined using a bioluminescence assay (Molecular Probes, Eugene, OR) utilizing recombinant firefly luciferase and its substrate, d-luciferin and following manufacturer's instructions. The luminescence was read in a TD-20/20 luminometer (Turner Designs, Sunnyvale, CA) at 560 nm. The ATP values were calculated using an ATP standard curve. The protein concentrations of the lysates were estimated using the BCA assay and ATP was expressed as pmol per μg protein. All data are expressed as the mean ± SD of three experiments. Statistical significance was assessed by the two-sample t test (independent variable).

### NADPH Measurements

Cells were harvested, washed with ice cold PBS and homogenized in NADPH extraction buffer (EnzyFluo, Bioassay Systems, Hayward, CA). NADPH was then measured following manufacturer's instructions (Bioassay Systems) by reduction of the provided probe into a fluorescent product measured at λ_ex/em_ of 530/585 nm. Values were compared with a standard curve. All data are expressed as the mean ± SD of three experiments. Statistical significance was assessed by the two-sample t test (independent variable).

### Apoptosis Assay

Cells were stained with FITC-labeled annexin-V and propidium iodide following the manufacturer's protocol (BD Biosciences, San Diego, CA). Briefly, cells were detached and 100,000 cells per sample were pelleted by centrifugation at 1500 rpm for 5 minutes and washed X1 with PBS. The cells were then resuspended in 1X binding buffer, annexin-V/FITC and/or propidium iodide added and were incubated in the dark at room temperature for 15 minutes. 1X binding buffer was added to increase volume and 10,000 events were counted for each sample using the appropriate filters for FITC and PI detection (BD FACSCalibur, San Jose, CA). Data was analyzed using FlowJo software (TREE STAR Inc, San Carlos, CA). Results were calculated as the average of triplicate samples ± SD.

### NMR Experiments

Cells treated with siRNA were grown in media containing uniformly labeled ^13^C-glucose (2 gm/L, Cambridge Isotopes Laboratories, Andover, MA) for 24 hours. Media samples were frozen in liquid nitrogen. Cells were counted and equal numbers of cells were pelleted, washed twice with cold PBS to remove adhering medium, and flash frozen in liquid nitrogen. The cell pellets and media samples were extracted with 10% ice-cold trichloracetic acid (TCA) followed by lyophilization. The extracts were redissolved in D_2_O containing 85 μM (media) or 95 μM (cells) DSS (2, 2-dimethyl-2-silapentane-5-sulfonate sodium salt) as both a chemical shift reference and as a concentration standard and loaded into 5 mm Shigemi tubes (Shigemi, Tokyo, Japan). Nuclear magnetic resonance (NMR) spectra were recorded at 14.1 T on a Varian Inova spectrometer at 20°C using a 90° excitation pulse as described previously [30]. 1D proton NMR spectra of extracts were recorded with a 2 sec acquisition time and a total recycle time of 5 sec, with presaturation of the residual HOD resonance for 3 seconds. For analyzing the cellular extracts and determining the positional enrichment with ^13^C we used 2D experiments (TOCSY and ^1^H{^13^C}-HSQC), and quantified the ^13^C satellite and ^12^C peaks by volume integration in the TOCSY using established assignment protocols [30, 31]. The NMR experiments were carried out in the Structural Biology Program's NMR Core at the University of Louisville James Graham Brown Cancer Center.

### Mouse Studies

H460 cells stably transfected with either PFKFB4 or control shRNA were collected from exponential growth phase culture. Cells were washed twice, re-suspended in PBS (5 × 10^7^ cells/ml) and groups of 10 BALB/c athymic female mice were injected s.c. with 100 μL (5 × 10^6^ cells) of the cell suspension. The tumors were followed from the time of appearance until the end of the experiment. Tumor masses were determined in a blinded fashion with Vernier calipers according to the following formula: weight (mg) = (width, mm)^2^ × (length, mm)/2 [32]. All data are expressed as the mean ± SD of two experiments. Statistical significance was assessed by the two-sample t test (independent variable). At the end of the experiment, the animals were euthanized and tumors removed and fixed in 10% formaldehyde. Sections were stained with hematoxylin/eosin and for immunohistochemistry as described below. At the end of the experiment, subsets of xenograft-bearing mice (n=4) were injected i.p. with 2-[^18^F]-fluoro-2-deoxyglucose (^18^F-FDG, 150μCi, 100 μL in H_2_O) and, after 45 min, were anesthetized with 2% isoflurane in oxygen and transferred to a R-4 Rodent Scanner (CTI Concorde Microsystems) micro-positron emission tomograph to capture images. Regions of interest in the tumors and cerebellum were quantified in quadruplicate and are expressed as the mean ± SD of the ratio of tumor:cerebellar FDG uptake. Animal experiments were carried out in accordance with established practices as described in the National Institutes of Health Guide for Care and Use of Laboratory Animals and were approved by the University of Louisville Institutional Animal Care and Use Committee.

### Immunohistochemistry

Five μm mounted sections of formalin-fixed and paraffin-embedded tumor tissue were deparaffinized with xylene. Epitope retrieval was carried out using citrate buffer in a 2100 Retriever (PickCell Laboratories). The sections were blocked with 10% goat serum for 1 hour, then incubated with primary antibody against PFKFB3, PFKFB4, carbonic anhydrase IX (Proteintech) or cleaved caspase-3 (Cell Signaling, Danvers, MA) overnight, followed by an HRP-linked goat anti-rabbit secondary antibody (1:300, Pierce Biotechnology). The sections were developed with 3,3′-diaminobenzidine tetrahydrochloride (DAB, Vector Laboratories, Burlingame, CA) for 2 min, nuclei counterstained with Mayer's hematoxylin (Sigma-Aldrich) for 2 min and coverslips attached with Permount (Fisher Scientific, Fair Lawn, NJ). Slides were scanned using a ScanScope XT Digital Slide Scanner (Aperio), data analyzed with the positive pixel count algorithm (ImageScope, Aperio) and a minimum of 5 fields (20x magnification) were quantified for each tumor section. The data is depicted as % positive pixels/total pixels ± SD.

## References

[1] Yalcin A, Telang S, Clem B, Chesney J (2009). Regulation of glucose metabolism by 6-phosphofructo-2-kinase/fructose-2,6-bisphosphatases in cancer. Exp Mol Pathol.

[2] Okar DA, Manzano A, Navarro-Sabate A, Riera L, Bartrons R, Lange AJ (2001). PFK-2/FBPase-2: maker and breaker of the essential biofactor fructose-2,6-bisphosphate. Trends Biochem Sci.

[3] Van Schaftingen E, Hue L, Hers HG (1980). Fructose 2,6-bisphosphate, the probably structure of the glucose- and glucagon-sensitive stimulator of phosphofructokinase. Biochem J.

[4] Van Schaftingen E, Hue L, Hers HG (1980). Control of the fructose-6-phosphate/fructose 1,6-bisphosphate cycle in isolated hepatocytes by glucose and glucagon. Role of a low-molecular-weight stimulator of phosphofructokinase. Biochem J.

[5] el-Maghrabi MR, Correia JJ, Heil PJ, Pate TM, Cobb CE, Pilkis SJ (1986). Tissue distribution, immunoreactivity, and physical properties of 6-phosphofructo-2-kinase/fructose-2,6-bisphosphatase. Proc Natl Acad Sci U S A.

[6] Manzano A, Perez JX, Nadal M, Estivill X, Lange A, Bartrons R (1999). Cloning, expression and chromosomal localization of a human testis 6-phosphofructo-2-kinase/fructose-2,6-bisphosphatase gene. Gene.

[7] Sakata J, Abe Y, Uyeda K (1991). Molecular cloning of the DNA and expression and characterization of rat testes fructose-6-phosphate,2-kinase:fructose-2,6-bisphosphatase. J Biol Chem.

[8] Sakakibara R, Kato M, Okamura N, Nakagawa T, Komada Y, Tominaga N, Shimojo M, Fukasawa M (1997). Characterization of a human placental fructose-6-phosphate, 2-kinase/fructose-2,6-bisphosphatase. J Biochem.

[9] Sakakibara R, Uemura M, Hirata T, Okamura N, Kato M (1997). Human placental fructose-6-phosphate,2-kinase/fructose-2,6-bisphosphatase: its isozymic form, expression and characterization. Biosci Biotechnol Biochem.

[10] Minchenko OH, Opentanova IL, Ogura T, Minchenko DO, Komisarenko SV, Caro J, Esumi H (2005). Expression and hypoxia-responsiveness of 6-phosphofructo-2-kinase/fructose-2,6-bisphosphatase 4 in mammary gland malignant cell lines. Acta Biochim Pol.

[11] Minchenko OH, Ochiai A, Opentanova IL, Ogura T, Minchenko DO, Caro J, Komisarenko SV, Esumi H (2005). Overexpression of 6-phosphofructo-2-kinase/fructose-2,6-bisphosphatase-4 in the human breast and colon malignant tumors. Biochimie.

[12] Minchenko OH, Ogura T, Opentanova IL, Minchenko DO, Ochiai A, Caro J, Komisarenko SV, Esumi H (2005). 6-Phosphofructo-2-kinase/fructose-2,6-bisphosphatase gene family overexpression in human lung tumor. Ukr Biokhim Zh.

[13] Minchenko O, Opentanova I, Caro J (2003). Hypoxic regulation of the 6-phosphofructo-2-kinase/fructose-2,6-bisphosphatase gene family (PFKFB-1-4) expression in vivo. FEBS letters.

[14] Goidts V, Bageritz J, Puccio L, Nakata S, Zapatka M, Barbus S, Toedt G, Campos B, Korshunov A, Momma S, Van Schaftingen E, Reifenberger G, Herold-Mende C, Lichter P, Radlwimmer B (2012). RNAi screening in glioma stem-like cells identifies PFKFB4 as a key molecule important for cancer cell survival. Oncogene.

[15] Ros S, Santos CR, Moco S, Baenke F, Kelly G, Howell M, Zamboni N, Schulze A (2012). Functional metabolic screen identifies 6-phosphofructo-2-kinase/fructose-2,6-biphosphatase 4 as an important regulator of prostate cancer cell survival. Cancer Discov.

[16] Cohen JS, Lyon RC, Chen C, Faustino PJ, Batist G, Shoemaker M, Rubalcaba E, Cowan KH (1986). Differences in phosphate metabolite levels in drug-sensitive and -resistant human breast cancer cell lines determined by 31P magnetic resonance spectroscopy. Cancer Res.

[17] Imbert-Fernandez Y, Clem BF, O’Neal J, Kerr DA, Spaulding R, Lanceta L, Clem AL, Telang S, Chesney J (2014). Estradiol stimulates glucose metabolism via 6-phosphofructo-2-kinase (PFKFB3). J Biol Chem.

[18] Chesney J, Mitchell R, Benigni F, Bacher M, Spiegel L, Al-Abed Y, Han JH, Metz C, Bucala R (1999). An inducible gene product for 6-phosphofructo-2-kinase with an AU-rich instability element: role in tumor cell glycolysis and the Warburg effect. Proc Natl Acad Sci U S A.

[19] Navarro-Sabate A, Manzano A, Riera L, Rosa JL, Ventura F, Bartrons R (2001). The human ubiquitous 6-phosphofructo-2-kinase/fructose-2,6-bisphosphatase gene (PFKFB3): promoter characterization and genomic structure. Gene.

[20] Telang S, Yalcin A, Clem AL, Bucala R, Lane AN, Eaton JW, Chesney J (2006). Ras transformation requires metabolic control by 6-phosphofructo-2-kinase. Oncogene.

[21] Clem B, Telang S, Clem A, Yalcin A, Meier J, Simmons A, Rasku MA, Arumugam S, Dean WL, Eaton J, Lane A, Trent JO, Chesney J (2008). Small-molecule inhibition of 6-phosphofructo-2-kinase activity suppresses glycolytic flux and tumor growth. Mol Cancer Ther.

[22] Bobarykina AY, Minchenko DO, Opentanova IL, Moenner M, Caro J, Esumi H, Minchenko OH (2006). Hypoxic regulation of PFKFB-3 and PFKFB-4 gene expression in gastric and pancreatic cancer cell lines and expression of PFKFB genes in gastric cancers. Acta Biochim Pol.

[23] Minchenko A, Leshchinsky I, Opentanova I, Sang N, Srinivas V, Armstead V, Caro J (2002). Hypoxia-inducible factor-1-mediated expression of the 6-phosphofructo-2-kinase/fructose-2,6-bisphosphatase-3 (PFKFB3) gene. Its possible role in the Warburg effect. J Biol Chem.

[24] Minchenko O, Opentanova I, Minchenko D, Ogura T, Esumi H (2004). Hypoxia induces transcription of 6-phosphofructo-2-kinase/fructose-2,6-biphosphatase-4 gene via hypoxia-inducible factor-1alpha activation. FEBS Lett.

[25] Cordero-Espinoza L, Hagen T Increased Concentrations of Fructose 2,6-Bisphosphate Contribute to the Warburg Effect in Phosphatase and Tensin Homolog (PTEN)-deficient Cells. J Biol Chem.

[26] Garcia-Cao I, Song MS, Hobbs RM, Laurent G, Giorgi C, de Boer VC, Anastasiou D, Ito K, Sasaki AT, Rameh L, Carracedo A, Vander Heiden MG, Cantley LC, Pinton P, Haigis MC, Pandolfi PP Systemic elevation of PTEN induces a tumor-suppressive metabolic state. Cell.

[27] McArthur GA, Puzanov I, Amaravadi R, Ribas A, Chapman P, Kim KB, Sosman JA, Lee RJ, Nolop K, Flaherty KT, Callahan J, Hicks RJ (2012). Marked, homogeneous, and early [18F]fluorodeoxyglucose-positron emission tomography responses to vemurafenib in BRAF-mutant advanced melanoma. J Clin Oncol.

[28] Clem BF, O’Neal J, Tapolsky G, Clem AL, Imbert-Fernandez Y, Kerr DA, Klarer AC, Redman R, Miller DM, Trent JO, Telang S, Chesney J (2013). Targeting 6-phosphofructo-2-kinase (PFKFB3) as a therapeutic strategy against cancer. Mol Cancer Ther.

[29] Van Schaftingen E, Lederer B, Bartrons R, Hers HG (1982). A kinetic study of pyrophosphate: fructose-6-phosphate phosphotransferase from potato tubers. Application to a microassay of fructose 2,6-bisphosphate. Eur J Biochem.

[30] Telang S, Lane AN, Nelson KK, Arumugam S, Chesney J (2007). The oncoprotein H-RasV12 increases mitochondrial metabolism. Mol Cancer.

[31] Lane AN, Fan TW, Higashi RM (2008). Isotopomer-based metabolomic analysis by NMR and mass spectrometry. Biophysical Tools for Biologists.

[32] Taetle R, Rosen F, Abramson I, Venditti J, Howell S (1987). Use of nude mouse xenografts as preclinical drug screens: in vivo activity of established chemotherapeutic agents against melanoma and ovarian carcinoma xenografts. Cancer Treat Rep.

